# Genomic analysis of DNA repair genes and androgen signaling in prostate cancer

**DOI:** 10.1186/s12885-018-4848-x

**Published:** 2018-10-10

**Authors:** Kasey Jividen, Katarzyna Z Kedzierska, Chun-Song Yang, Karol Szlachta, Aakrosh Ratan, Bryce M Paschal

**Affiliations:** 1 Center for Cell Signaling, University of Virginia, Charlottesville, VA USA; 2 Center for Public Health Genomics, University of Virginia, Charlottesville, VA USA; 3 Department of Biochemistry and Molecular Genetics, University of Virginia, Charlottesville, VA USA; 4 Department of Public Health Sciences, University of Virginia, Charlottesville, VA USA

**Keywords:** Prostate cancer, Androgen receptor, DNA repair and DNA damage response

## Abstract

**Background:**

The cellular effects of androgen are transduced through the androgen receptor, which controls the expression of genes that regulate biosynthetic processes, cell growth, and metabolism. Androgen signaling also impacts DNA damage signaling through mechanisms involving gene expression and transcription-associated DNA damaging events. Defining the contributions of androgen signaling to DNA repair is important for understanding androgen receptor function, and it also has translational implications.

**Methods:**

We generated RNA-seq data from multiple prostate cancer lines and used bioinformatic analyses to characterize androgen-regulated gene expression. We compared the results from cell lines with gene expression data from prostate cancer xenografts, and patient samples, to query how androgen signaling and prostate cancer progression influences the expression of DNA repair genes. We performed whole genome sequencing to help characterize the status of the DNA repair machinery in widely used prostate cancer lines. Finally, we tested a DNA repair enzyme inhibitor for effects on androgen-dependent transcription.

**Results:**

Our data indicates that androgen signaling regulates a subset of DNA repair genes that are largely specific to the respective model system and disease state. We identified deleterious mutations in the DNA repair genes *RAD50* and *CHEK2*. We found that inhibition of the DNA repair enzyme MRE11 with the small molecule mirin inhibits androgen-dependent transcription and growth of prostate cancer cells.

**Conclusions:**

Our data supports the view that crosstalk between androgen signaling and DNA repair occurs at multiple levels, and that DNA repair enzymes in addition to PARPs, could be actionable targets in prostate cancer.

**Electronic supplementary material:**

The online version of this article (10.1186/s12885-018-4848-x) contains supplementary material, which is available to authorized users.

## Background

Prostate cancer remains the most commonly diagnosed cancer in men, with a lifetime risk of approximately 1 in 7 [1, 2]. Both cancer-associated events and the normal physiology of prostate involve signaling through the androgen receptor (AR) [3]. Indeed, clinical intervention based on androgen deprivation therapy (ADT), which reduces AR signaling, is a cornerstone of prostate cancer treatment. Resistance to ADT invariably develops and leads to the development of castrate resistant prostate cancer (CRPC) with associated morbidity and mortality. CRPC is characterized by changes in growth factor-, cell surface receptor-, and kinase-dependent signaling as well as gene expression that impact fundamental processes such as cell growth, motility, and DNA repair [4, 5]. Understanding how these changes occur, and defining actionable targets within the affected pathways, could expand the options for treating CRPC.

DNA repair enzymes have emerged as actionable targets for cancer, including prostate cancer. The data from clinical trials has shown that inhibiting the DNA repair enzyme PARP-1 in ovarian, breast, and prostate cancers improves outcome in patients that have genetic alterations in other components of the DNA repair machinery [6–8]. The success of PARP-1 inhibitors in this context suggests that new therapeutic opportunities might be revealed by understanding the interplay between genomic status and DNA repair pathways. There are also strong indications that the response to ionizing radiation (IR) can be influenced by androgen signaling. Thus, in pre-clinical models and in patients, ADT can confer radiosensitivity [9–11]. Finally, it has been shown that treating prostate cancer xenografts with inhibitors to AR (bicalutimide) and PARP-1 (Olaparib) inhibits tumor growth [12].

Pre-clinical models of prostate cancer, particularly a relatively small number of cell lines, are widely employed to study signal transduction and transcription, and to evaluate drug and IR sensitivities. A critical knowledge gap that may limit the interpretation and possibly the impact of data generated from these models is the genomic and transcriptomic state of the cells. To address this gap, we have performed whole genome sequencing (WGS) and RNA-seq on three prostate cancer lines. Combining the new data with publicly available data from human prostate cancers, we explored two important issues related to DNA damage signaling and repair. The first question was, do prostate cancer models harbor deleterious mutations in the DNA repair machinery. The second question was, does androgen signaling regulate expression of DNA repair machinery.

Our analysis revealed the presence of missense mutations in DNA repair genes in LNCaP, VCaP, PC3-AR, and RWPE-1 cells. Across these models, a total of 34 DNA damage response (DDR) genes were up-regulated, and 87 DDR genes were down-regulated in response to androgen. By co-expression network analysis, we found that expression of 25 DDR genes were altered in response to androgen treatment of cell lines. We also explored the interplay between DNA repair and AR-dependent transcription. Treating cells with the small molecular inhibitor, mirin, which inhibits the endonuclease MRE11, reduced AR-induced transcription. Our genomic and RNA-seq data should be useful for groups studying how the status of the DNA repair machinery influences properties such as drug sensitivity. The mirin effect on AR activity and cell growth suggests it might have utility as an inhibitor of prostate cancer cells.

## Methods

### Antibodies, reagents, and standard techniques

For immunoblotting experiments, lab-prepared AR hinge 3 (against AR residues 656 to 669: TQKLTVSHIEGYEC), Hsp70 (Stressgen), Hsp90β (GeneTex), lab-prepared FKBP51 (against FL protein), and Tubulin (Sigma Aldrich) were used. Secondary antibodies were IRDye-800 labeled antibodies (Rockland #610–732- 124, #610–132-121, and #611–732- 127), and Alexa Fluor-680 labeled secondary antibodies (Life Technologies #A21058 and #A10043). Standard immunoblotting procedures were conducted, images were detected on a fluorescent Odyssey imager, and analyzed using the provided LI-COR software.

For immunofluorescence, a lab prepared AR21 antibody (against AR residues 1 to 21: MEVQLGLGRVYPRPPSKTYRGC) was used while DAPI staining was used for nuclear detection. The secondary antibody was a Cy3-labeled anti-rabbit (Jackson Immunoresearch). Cells were grown on glass coverslips. Coverslips were prepared using standard immunofluorescence methods with a 15-min fixation (3.75% formaldehyde), washed with PBS, permeabilized for 5 min (0.2% triton X-100), and incubated with a 1-h block at room temperature (2% FBS and 2% BSA in PBS). Coverslips were then incubated in primary antibody (diluted in blocking buffer) overnight at 4 °C. Secondary antibodies (diluted in blocking buffer) were incubated for 1 h at room temperature. Images were obtained using a confocal microscope (Zeiss 800 LSM, Carl Zeiss) at 40×, 1.3 NA oil immersion objective and captured/processed using ZEN software (Carl Zeiss).

For immunoprecipitation, cells were lysed with cell lysis buffer (20 mM Tris-HCl pH 7.5, 50 mM NaCl, 0.5% Triton X-100, 5 mM EDTA, 2 mM DTT, and protease inhibitors) and clarified by centrifugation prior to incubation with Anti-FLAG M2 Affinity Gel (Sigma-Aldrich) at 4 °C. After a 4-h incubation, beads were washed with wash buffer (20 mM Tris-HCl pH 7.5, 50 mM NaCl, 0.1% Triton X-100, 0.1 mM EDTA, 2 mM DTT, and protease inhibitors). Standard SDS-PAGE loading buffer and procedures were used to separate proteins.

### Cell culture

LNCaP, VCaP, and PC-3 cells were kindly provided by Dr. Michael Weber (University of Virginia) and were purchased originally from ATCC. VCaP cells were grown in DMEM supplemented with 10% FBS and 1% antibiotic/antimycotic. LNCaP and PC-3 cells were grown in RPMI supplemented with 5% FBS and 1% antibiotic/antimycotic. PC3-AR cells were made by stable lenti-viral infection of full length AR using a pWPI-GFP-FLAG-AR plasmid in which the GFP portion was swapped with the antibiotic selectable hygromycin resistance gene. RWPE-1 cells were obtained from Dr. Daniel Gioeli and cultured in Keratinocyte SFM supplemented with the provided EGF and BPE factors. All cells were incubated at 5% CO_2_ and 37 °C.

### Cell growth/survival assays and cell cycle analysis

Cells were seeded onto a 96-well format for 1 day. Media was exchanged and supplemented with indicated concentrations of inhibitors for 72 h. Alamar blue dye (Promega, #G808A) was added (10% of total volume) for ~ 6 h and measured with a fluorescent plate reader according to manufacturer’s recommendations. Technical replicates of at least 4 measurements were averaged. The data was normalized by removing the background signal and rescaling the values so that the vehicle condition was 100%. Values were plotted using Prism software and IC_50_ values were calculated with non-linear regression based on the log transformed data.

Cell cycle measurements were determined using the FITC-conjugated BrdU Flow Kit (BD, #559619) where we stained and performed flow cytometry of asynchronous cells according to the manufacturer’s instructions and in reference to Benamar et al. (2016) [13]. In brief, cells were pulsed with BrdU for 1 h. Cells were washed and harvested using trypsin. After a PBS wash, cells were fixed. Prior to staining with 7-AAD and anti-BrdU antibody, cells were permeabilized. Cells were resuspended in a 1-mL solution and more than 10,000 cells were measured using the Cytek modified BD FACSCalibur provided by the Flow Cytometry Core Facility at the University of Virginia. Data was analyzed using the FlowJo and ModFit software packages.

### Gene expression analysis

Prostate cancer cells were plated in the corresponding phenol-free based media supplemented with charcoal-stripped serum (Gemini) for 48–72 h. Synthetic androgen, R1881 (Sigma Aldrich), was typically added for 12 h at a concentration of 2 nM.

For real time quantitative PCR (RT-qPCR) experiments, RNA was extracted using standard TRIzol (Thermo Fisher Scientific) methods. cDNA was prepared using BioRad iScript reagents and expression was detected using SensiMix Sybrgreen reagents, all according to manufacturer’s instructions. Technical replicates were averaged and normalized to the *GUS* housekeeping gene. Experiments are representatives of at least 3 experiments.

The following primers were used at a final concentration of 200 nM:

- *FKBP5* Forward: 5’-AGGAGGGAAGAGTCCCAGTG-3’
- *FKBP5* Reverse: 5’-TGGGAAGCTACTGGTTTTGC-3′

- *ABCC4* Forward: 5’-GGCAGTGACGCTGTATGG-3’
- *ABCC4* Reverse: 5’-CGCCAGGTCTGACAGTAAAG-3′

- *GUS* Forward: 5’-CCGACTTCTCTGACAACCGACG-3’
- *GUS* Reverse: 5’-AGCCGACAAAATGCCGCAGACG-3′

- *PSA* Forward: 5’-TGGTGCATTACCGGAAGTGGATCA-3’
- *PSA* Reverse: 5’-GCTTGAGTCTTGGCCTGGTCATTTC-3′

- *TMPRSS2* Forward: 5’-GGACAGTGTGCACCTCAAAGAC -3’
- *TMPRSS2* Reverse: 5’-TCCCACGAGGAAGGTCCC -3′

- *NCAPD3* Forward: 5’-TGACACAGTGTGGGAACTGG -3’
- *NCAPD3* Reverse: 5’-TAAAGCCCAGCGGCATGAAG -3′

- *p21* Forward: 5’-ATGTGTCCTGGTTCCCGTTTC -3′
- *p21* Reverse: 5′- CATTGTGGGAGGAGCTGTGA -3′

- *SOCS2* Forward: 5′- CTTGAGCCCTCCGGGAAT -3’
- *SOCS2* Reverse: 5′- TCCCCAGTACCATCCTGTCTG -3′

- *HOMER2* Forward: 5′- CGTCACAGAAGTTTGGGCAGTG -3’
- *HOMER2* Reverse: 5′- CTTGGCAGCTTCTTTCACCTCC -3′

- *EAF2* Forward: 5′- CCTTCCACACTGTGCGCTATGA -3’
- *EAF2* Reverse: 5′- GGCAGAGTTATGGTCACCTGTTC -3′

- *PIAS1* Forward: 5′- ACAGTGCGGAACTAAAGCAAA -3’
- *PIAS1* Reverse: 5′- AACCGCCGCCTATAGAGTTC -3′

For RNA-sequencing experiments, the Qiagen RNeasy kit was used to extract RNA. Library preparation and sequencing was performed by Hudson Alpha. Briefly, RNA integrity and concentration were assessed by a fluorometric assay, indexed libraries were made using the standard polyA method, quality control was used to determine size and concentration, and samples were sequenced using Illumina HiSeq 2500 at a depth of 250 million × 50-bp paired-end reads. Reads were aligned to the hg38 genome (ENSEMBL GRCh38.89) using STAR (release v. 2.5) [14]. Counts were generated using HTSeq (release v. 0.6) [15]. DESeq2 R package was used to determine normalized counts [16]. Genes with low counts were eliminated (≤ 10 in all conditions), and definitions of differential genes are described in the figure legends.

For weighted gene co-expression network analyses (WGCNA), we filtered the count matrix to remove genes with low read counts (where sum of reads in all samples < 1). We then applied variance stabilizing transformation to the remaining data resulting in homoskedastic counts normalized with respect to library size. Unsupervised clustering was performed with WGCNA [17, 18]. Briefly, a network was constructed using biweight midcorrelation as the measure of similarity between genes with β equal to 5. Modules were identified by applying hierarchical clustering (average method) to distance calculated from signed topological overlap matrix and the tree was cut with cutreeDynamic using the following parameters: minimum module size equal to 30 and hybrid method. Next, the modules were merged if the distance between them was equal to less than 0.25, resulting in 15 modules. We then calculated the eigengene for those 15 modules and created a gene list representing each module by filtering the genes based on gene significance and intra-modular connectivity. Modules were subsequently described by overrepresented pathways using Enrichr. Gene Set Enrichment Analysis (GSEA) was performed on pre-ranked gene list that was generated by assigning a value to each gene that was equal to log of *p*-value multiplied by the negative sign of the fold change (rank = − 1 * sign(FC) * log(*p*-value)). Gene sets used for analysis with GSEA included the MSigDB hallmark gene sets [19] as well as a curated DDR gene set of 450 genes.

### Data sources

Androgen-dependent and androgen-independent microarray data was downloaded using Gene Expression Omnibus (GEO) data repository (GSE847) [20]. We restricted our analysis to a curated list of DNA repair genes [21]. To confidently assess the expression of DNA repair genes, we filtered the normalized data by re-scoring negative intensities and values with “absent” detection calls [22]. Fold changes between hormone insensitive and hormone sensitive xenograft pairs were calculated, and fold changes ≥1.5 and ≤ 0.667 were defined as an alteration.

The publicly available Memorial Sloan-Kettering Cancer Center (MSKCC) prostate adenocarcinoma (PRAD) dataset was downloaded from cBioPortal [23, 24]. Clinical and expression data for a total of 181 primary prostate tumors and 37 metastatic tumors were provided by Taylor et al. (2010) [25]. We limited our analysis to the 450 expert-curated DNA damage/DNA repair gene set from Pearl et al. (2015) [21] and tested the association of the normalized expression for various clinical parameters using the Kruskal-Wallis test (*p*-value ≤1E-5). To generate the heatmap, we used “mRNA Expression Z-Scores vs Normals” data that was normalized and analyzed by cBioPortal. We plotted those genes found to be significantly associated with disease status using ComplexHeatmap.

In order to test a significance based on DNA damage response ontologies, we used Fisher’s exact test (fisher.test() in R v. 3). We generated unions of genes from each of the 125 ontology pathways described by Pearl et al. (2015) [21]. We then classified the genes from each of our analyses (cell line, xenograft, patient metastases, or all groups) based on their presence or absence in the ontology group. Returned *p*-values for each ontology group were plotted as a function of the -log10 value.

ChIP-sequencing analysis was derived from GSE28126. LNCaP and VCaP data was aligned to the hg38 reference genome using bowtie2 [26]. Peaks were called using macs2 [27]. To determine androgen-induced peaks, R1881 treated samples were analyzed as the “treatment” while untreated samples were analyzed as the “control”. Peaks were annotated to the closest gene using bedtools [28].

### Additional image processing information

Figures related to RT-qPCR and S-plots were generated using Microsoft Excel and graphed using GraphPad Prism (GraphPad Software, La Jolla, CA). GSEA (Broad Institute, Inc., release v. 3.0) was used to calculate gene set enrichments [29, 30]. The data was replotted using R. ComplexHeatmap and Python (matplotlib.pyplot.imshow) were used to graph heatmap data. Lollipops (release v. 1.3.2) was used to plot protein mutations [31]. All figures were assembled with Adobe Illustrator.

### Detecting genomic variants

For LNCaP, VCaP, and PC3-AR cell lines, genomic DNA was prepared using the Qiagen DNeasy kit. Libraries were prepared and samples were sequenced by Hudson Alpha. Sequenced DNA data from RWPE-1 was kindly provided by Dr. Anindya Dutta (University of Virginia). Generation of sequenced RNA datasets were described above. To provide additional coverage for RNA transcripts, the sequenced reads from both the control and the androgen-treated samples were merged for each respective cell line. The RWPE-1 RNA-seq sample was derived by merging the following publicly available datasets: SRR1282953, SRR2919800, and SRR2919800.

DNA was aligned using BWA [32, 33] to the hg19 reference sequence. Corresponding RNA-seq was aligned using STAR [14] to the hg19 reference sequence. Aligned reads were subsequently filtered and processed using GATK Haplotype Caller [34]. Variants were limited to the following 18 DNA repair genes: *PARP1*, *PARP2*, *ERCC3*, *ATR*, *ATM*, *RAD50*, *RAD51*, *MRE11*, *NBN*, *CHEK1*, *CHEK2*, *MLH3*, *PALB2*, *FANCA*, *BRCA1*, *BRCA2*, *HDAC2*, and *PRKDC*. Allelic frequency for each variant were compared to the 1000 Genomes Project [35] and the NHLBI GO Exome Sequence Project [36]. COSMIC identification numbers [37, 38] and prior reports for each variant was verified through a literature search [39]. Deleterious mutations were predicted in silico by Scale-invariant feature transform (SIFT) [40], fitness consequence (fitCons) [41], Combined Annotation-Dependent Depletion (CADD) [42], and Polymorphism Phenotyping (PolyPhen) [43].

## Results

### Prostate cancer cell line models contain potential deleterious mutations in the DNA repair machinery

**Fig. 1:**
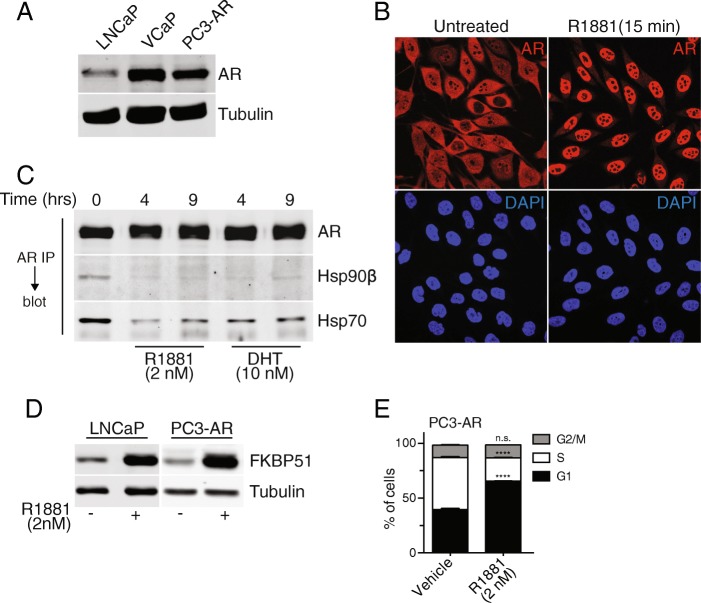
Characterization of PC3 cells stably transduced with WT AR. **a** AR protein expression level in PC3-AR cells compared to LNCaP and VCaP cells. **b** Immunofluorescence localization of AR in PC3-AR cells before and after treatment with synthetic androgen (2 nM R1881, 15 min), which induces nuclear import of AR. **c** AR complexes isolated by immunoprecipitation and examined for Hsp90β and Hsp70 content, which is released by androgen. **d** Immunoblot showing androgen-induced expression of FKBP51 in LNCaP and PC3-AR cells after an overnight treatment of 2 nM R1881. **e** Androgen-induced G1 cell cycle arrest after a 12 h R1881 treatment

We set out to define the state of the DNA repair machinery in prostate cancer cell lines, including how it is influenced by androgen signaling. A potential difficulty of comparing androgen signaling across prostate cancer cells is - depending on the line - the AR is mutated, alternatively spliced, and expressed at different levels, all of which could affect the transcriptional output measured in response to androgen [44, 45]. For example, the most widely used prostate cancer cell line, LNCaP, carries a mutation in the ligand binding domain that affects activity. To help address this issue, we generated a line using PC3 cells, in which WT AR was re-introduced. PC3 cells are a metastatic, AR-negative prostate cancer line that is arguably the most aggressive prostate cancer line used in the laboratory. AR protein expression in PC3-AR cells is similar to the level in VCaP and LNCaP and shows efficient translocation into the nucleus 15 min after the addition of the synthetic androgen, R1881 (Fig. 1a-b). Like other prostate cancer lines, AR re-expressed in PC3 cells displays R1881- and DHT-induced release of Hsp90 and Hsp70, which reflects conformational changes induced by androgen binding to the AR ligand binding domain (Fig. 1c). Androgen-induced transcription and translation of the *FKBP5* gene, which is directly regulated by AR [46] and widely used as a readout of AR activity, generates comparable levels of FKBP51 protein detected by immunoblotting in PC3-AR and LNCaP cells (Fig. 1d). These data indicate that AR stably reintroduced into PC3 cells responds to androgen, activates endogenous gene expression, and can be used as a model to study WT AR function in prostate cancer cells. We also determined that R1881 treatment of PC3-AR cells increases the fraction of cells in G1 from 39 to 65% (Fig. 1e). This property is not unique to PC3-AR cells, as LNCaP show a biphasic growth response and undergo senescence in response to 1 nM R1881 [47, 48].

To define genomic alterations in DNA repair genes in prostate cancer cells, we performed whole-genome sequencing of PC3-AR, LNCaP, and VCaP cells to obtain an average coverage of ~ 30-fold. We also used a dataset generated previously from RWPE-1 cells. We focused on the mutational status of 18 DNA repair genes (*PARP1*, *PARP2*, *ERCC3*, *ATR*, *ATM*, *RAD50*, *RAD51*, *MRE11*, *NBN*, *CHEK1*, *CHEK2*, *MLH3*, *PALB2*, *FANCA*, *BRCA1*, *BRCA2*, *HDAC2*, *PRKDC*). Twelve of these DNA repair genes display genome-level alterations in prostate cancer that are associated with positive outcome in response to Olaparib treatment [6]. Six additional genes encoding other core components of the DNA repair machinery were also included [49]. We aligned the sequences to the b37 + decoy reference sequence, removed the putative PCR duplicates, and used the GATK Haplotyper pipeline [34] to identify single-nucleotide variants (SNV) in the DNA repair gene set across the four cell lines. From our analyses of the 18 DNA repair genes, we detected 12 missense mutations in the RWPE-1 cell line, 9 missense mutations in the VCaP cell line, 2 missense mutations in the PC3-AR cell line, and 22 missense mutations in the LNCaP cell line (Additional file 1**:** Table S1).

To address whether these mutations could be common alterations within the human population, we annotated the variants with observed allele-frequencies in European populations from the 1000 Genomes Project [35] and the NHLBI GO Exome Sequencing Project [36]. We filtered the missense mutations to identify those that have a reported allele frequency < 0.1. Four mutations, BRCA1 (D693N), BRCA2 (R2034C), PARP-1 (P377S), and PARP-1 (V762A) in the RWPE1 cell line passed the threshold. Three mutations in the VCaP cell line, ATM (F858 L), ATM (P1054R), and DNA-PK (R2899C) had lower frequencies than 0.1 while only PARP-1 (S383Y) was found to have a lower allelic frequency than 0.1 in the PC3-AR cell line. In our analysis, the LNCaP cell line contained the most missense and frameshift mutations including ATR (K297 N), ATR (K1482R), CHEK2 (E239*), CHEK2 (T387 N), DNA-PK (N2669 V), DNA-PK (A3417T), ERCC3 (A740T), ERCC3 (R391W), FANCA (L684P), HDAC2 (A62V), MLH3 (K274I), MLH3 (I541V), NBS1 (D95N), PARP-1 (E547G), RAD50 (K608 N), and RAD50 (L719 fs*15). A literature and COSMIC search revealed that several of these mutations have been detected in other model systems and other cancer subtypes, e.g. PARP-1 (V377S), ATR (K297 N), FANCA (G809 N), and MLH3 (I541V) (Additional file 1: Table S1).

To determine whether the variants are expressed, we performed RNA-seq on the respective prostate cancer lines. We aligned the RNA-seq reads using STAR aligner, removed the putative PCR duplicates, used the SplitNCigarReads tool to partition the reads into smaller sequences representing segments beside/between splicing events, and then used the GATK Haplotyper to identify SNVs validating the DNA variant calls (Additional file 2**:** Table S2, “RNA-seq SNV detected”). Of note, the RNA-seq reads are limited by the expression of the gene and the location within the exon. In particular, reads that fall on the 3′ and 5′ ends of the exon will not be detected by this approach. We determined whether the SNV was detected by RNA-seq and if the corresponding exon/intron was expressed by RNA-seq (Additional file 2: Table S2, “SNV expressed” and “Exon/Intron expressed”, respectively). Using these criteria, we confirmed the following missense mutations are encoded at the mRNA level: (1) BRCA1 (D693N), BRCA2 (R2034C), PARP-1 (P377S), and PARP-1 (V762A) in RWPE-1 cells; (2) ATM (F858 L), ATM (P1054R), and DNA-PK (R2899C) in VCaP cells; (3) PARP-1 (S383Y) in PC3-AR cells; (4) ATR (K297 N), CHEK2 (T387 N), ERCC3 (A740T), ERCC3 (R391W), HDAC2 (A62V), MLH3 (I541V), and NBS1 (D95N) in LNCaP cells.

**Fig. 2:**
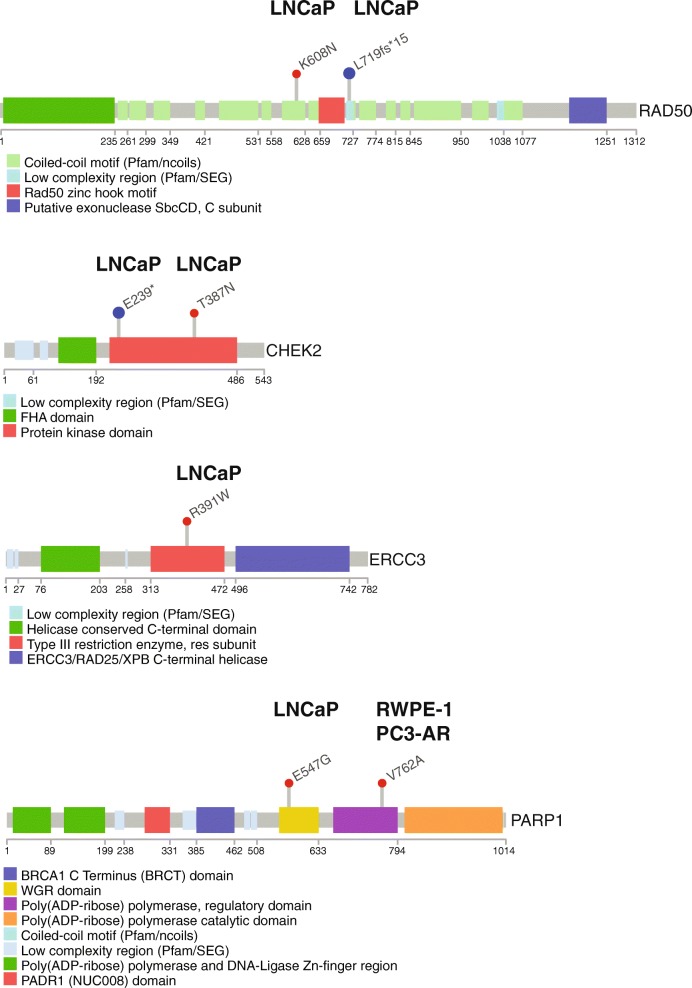
Commonly used prostate cell lines contain potentially deleterious mutations. Diagrams showing the positions (lollipops) of deleterious mutations in DNA repair proteins. Shown are the deleterious mutations found in RWPE-1, LNCaP, and PC3-AR cells. Red dots correspond to missense mutations and blue dots correspond to missense mutations projected to result in protein truncation

To determine if any of the genetic alterations are potentially deleterious, we used the following in silico–based methods: SIFT [40], fitCons [41], CADD [42], and PolyPhen [43]. These methods use algorithms to determine the likely impact of the mutation on the protein and are based on a number of criteria including sequence homology and relationships of a given alteration to essential protein domains. Two potential deleterious candidates - CHEK2 (E239*) and RAD50 (L719 fs*15) - were both found in LNCaP cells (Additional file 2: Table S2, Fig. 2). These mutations would result in a premature termination and in C-terminal truncations. In silico analyses also detected missense mutations in *PARP1*, *CHEK2*, *RAD50*, and *ERCC3* that would likely influence protein function based on where these alterations fall within the respective protein domain (Additional file 2: Table S2, Fig. 2). These results reveal that similar to the genetic alterations reported in CRPC patient samples, the cell line models contain deleterious mutations in DNA repair genes associated with the response to PARP inhibition. Thus, our data from these models, and particularly LNCaP cells, provides a baseline for exploring the interplay between DNA repair genes and therapeutics.

### Activation of AR generates shared and cell line-specific transcription programs

**Fig. 3:**
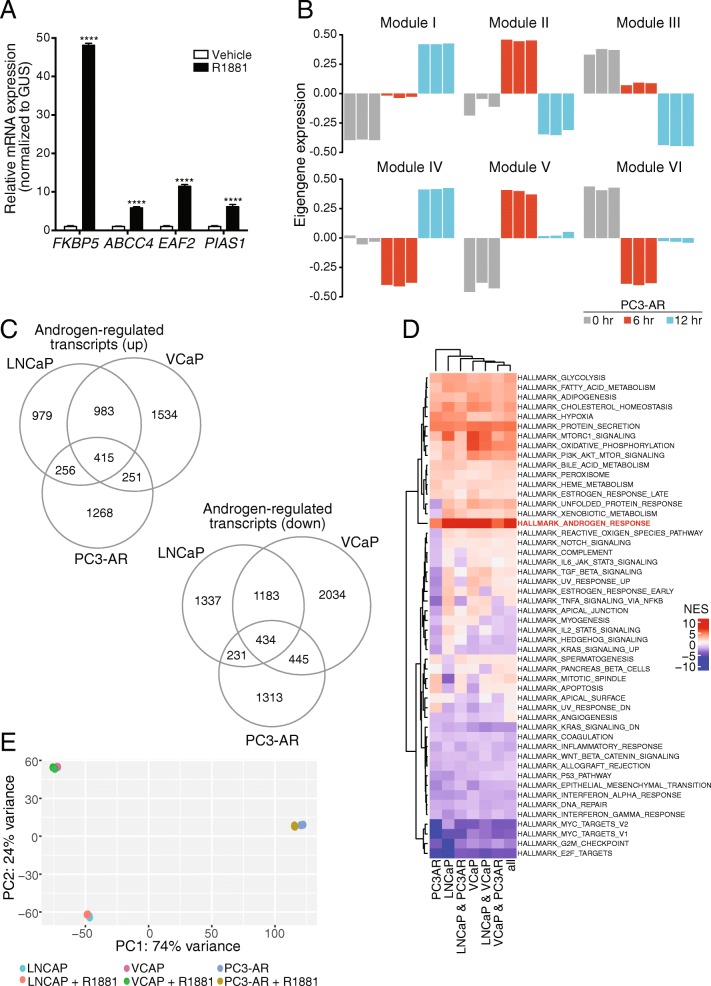
Androgen-dependent transcriptional responses of prostate cancer cell lines. **a** RT-qPCR shows androgen treatment (2 nM R1881, 12 h) induces *FKBP5*, *ABCC4*, *EAF2*, and *PIAS1* expression in PC3-AR cells. **b** Eigengene expression plots from WGCNA of PC3-AR RNA-sequencing. **c** Venn diagram of all differentially expressed transcripts in PC3-AR, LNCaP, and VCaP cell lines after 2 nM R1881 androgen treatment. Differential fold change was set to the following parameters: > 1.5-fold or < 0.67-fold; adj. *p* ≤ 0.01. LNCaP and VCaP treatments were conducted for 24 h while PC3-AR data was taken from a 12 h treatment. **d** Heatmap displaying normalized enrichment scores (NES) generated from a pre-ranked Gene Set Enrichment Analysis (GSEA) of androgen-treated prostate cancer cell lines. Standard GSEA methods were limited to the MSigDB hallmarks. **e** Principal component analysis (PCA) plot of mRNA expression data generated from RNA-sequencing. Data derived from LNCaP, VCaP, and PC3-AR cells before and after androgen treatment

Androgen signaling has been shown to alter the expression of DNA repair genes [9, 50]. To address whether this might be a general feature of prostate cancer cells, RNA-seq data that we generated from the PC3-AR, LNCaP, and VCaP lines was used to characterize the effects of androgen. Our initial focus was on the PC3-AR line. Using RT-qPCR, we found that androgen treatment (R1881, 2 nM) of PC3-AR cells for 12 h resulted in activation of AR target genes, including *FKBP5*, *ABCC4*, *EAF2*, and *PIAS1* (Fig. 3a). Transcripts for *PSA* and *TMPRSS2*, which are commonly used as readouts for androgen activation of transcription, were not detected in the presence or absence of androgen. To further characterize the androgen response in PC3-AR cells, we treated the cells with R1881 and harvested cells at 6 and 12 h time points for RNA-seq. Using the RNA-seq data, we employed co-expression networks to understand the broad patterns of expression changes in this cell line in response to androgen treatment. We used the WGCNA package [17, 18] which uses correlation-based inference methods to define gene-gene relationships, followed by construction of a network where each node represents a gene and each edge represents the presence and the strength of the co-expression relationship between the genes it connects. “Modules” in this case refers to genes that show a similar pattern of change with time and are identified using hierarchical clustering. Each gene is assigned a kME value that is a measure of the strength of connection of the gene with the module, and each module can be summarized using one representative gene called the “eigengene”.

We applied WGCNA to the PC3-AR RNA-seq dataset requiring a minimum module size of 30 genes. We initially identified 79 modules, but after filtering to limit the modules to (a) those with a membership greater than 100 genes and (b) those where the three replicates from the same time-point exhibit similar expression profiles, we were left with 6 modules (Fig. 3b). For each of the modules, we ranked the genes using their kME values. We then used Gene Set Enrichment Analysis (GSEA) to determine the hallmark gene-sets from MSigDB that were over-represented at the top of the list using an FDR threshold of 0.25 (Additional file 3: Table S3). The genes from modules I, IV and VI all demonstrated positive associations with the 12 h androgen time point relative to the untreated sample and were all significantly enriched for the “HALLMARK_ANDROGEN_RESPONSE”, a collection of genes from the MSigDB collection that define the androgen response [19].

We next compared the RNA-seq data from PC3-AR cells (12 h R1881) to RNA-seq data from VCaP and LNCaP cells (24 h R1881). We used DESeq2 [16] to identify differentially expressed genes (adjusted *p*-value < 0.05) in response to androgen treatment within and across the three cell lines. In response to R1881 treatment, PC3-AR cells had 2,190 transcripts that were upregulated and 2,423 transcripts that were downregulated, LNCaP cells had 2,633 transcripts that were upregulated and 3,185 transcripts that were downregulated, and VCaP cells had 3,183 transcripts that were upregulated and 4,096 transcripts that were downregulated (Fig. 3c). Notably, the androgen-controlled gene expression patterns from all three cell lines, including the PC3-AR cell line, was found to be strongly enriched for the “HALLMARK_ANDROGEN_RESPONSE” and the “HALLMARK_E2F_TARGETS”, in a positive and negative manner, respectively (Fig. 3d**,** Additional file 4**:** Figure S1). From principal components analysis, we found that the replicates within each group were highly similar and that most of the variability in the data is explained by the differences in the cell lines, which dominate over the effects of androgen (Fig. 3e). Overall, these results are in agreement with other reports showing that ligand activation of nuclear hormone receptors generates shared and cell line-specific transcription programs [51]. Multiple factors and pathways likely contribute to the differences observed in the cell lines, and these may include specific forms and expression levels of AR.

### Expression signatures of the DNA repair and DNA damage response genes in prostate cancer cell lines

The Sawyers group explored how ADT increases the radiosensitivity of CRPC, and reported that AR transcriptional output is associated with the expression of DNA repair genes [50]. GSEA was used to define an AR-associated DNA repair gene set in human tumors (144 genes) of which a subset (32 genes) are AR target genes. In light of these observations, we set out to understand if androgen regulation of DNA repair genes is a common feature of prostate cancer cells.

**Fig. 4:**
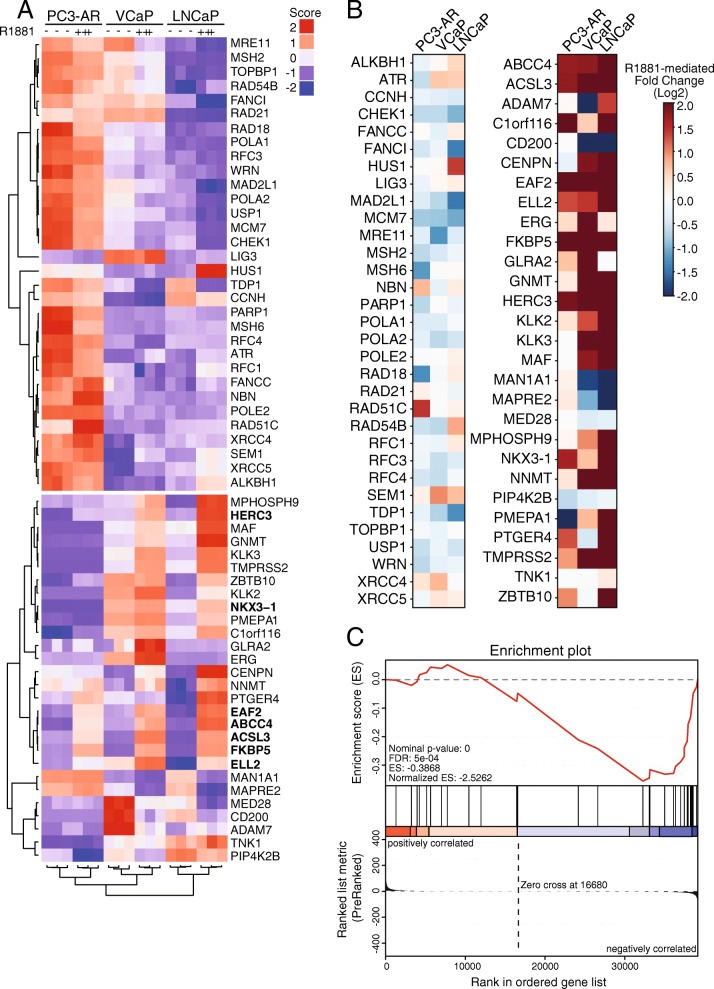
Reduced expression of multiple DNA repair genes occurs in response to androgen treatment. **a** Heatmap of DNA damage repair genes in prostate cancer cell line models. RNA-sequencing from untreated and androgen-treated cells were Z-score normalized for the 32 DNA damage repair genes reported by Polkinghorn et al. (2013) [50] and 28 control AR-target genes reported by Hieronymus et al. (2006) [52]*.* Genes in bold indicate differential regulation across all three cell lines. **b** Heatmap of DESeq2 log2 fold changes of DNA damage repair genes in prostate cancer cell line models. **c** GSEA of the RNA-seq data from prostate cancer lines, limited to the 32- DNA repair gene set signature

We used the 32 DNA repair gene set published by Polkinghorn et al. (2013) [50] to query our RNA-seq data and found that most of these genes were not significantly altered by androgen treatment in the three cell models (adjusted *p*-value < 0.05) (Fig. 4a). *HUS1* and *RAD51C* were two genes that were increased in LNCaP and PC3-AR, respectively. We found a total of seven genes underwent a significant reduction in response to androgen treatment (*MRE11*, *FANCI*, *RAD18*, *MAD2L1*, *MCM7*, *TDP1*, *MSH6*), though this effect did not extend to all three cell lines. From a heatmap of the regularized-log (rlog) transformed mRNA counts and androgen-mediated fold changes of 28 characterized well-characterized androgen-regulated genes [52] (Fig. 4a-b), it is clear that androgen induces gene expression changes in all three cell lines under our growth conditions.

The hierarchical clustering of the 32 DNA repair genes (Fig. 4a) based on a correlation distance and complete linkage identified 16 genes that showed a weak decrease in expression in response to androgen across all three cell lines. We repeated the DESeq2 analysis, treating the cell lines as biological replicates adjusting for the cell-line specific effects. In this analysis, we detected differential expression in response to androgen in 21 of the 32 genes (adjusted *p*-value < 0.05) - however, 17 of the 21 genes showed a decrease in expression (data not shown). This finding was supported by a GSEA, where we found a significant enrichment for genes that undergo an androgen-dependent decrease in expression (Fig. 4c). We found that 18 of the 32 genes formed the leading edge and account for the majority of the gene set enrichment signal (Fig. 4c).

**Fig. 5:**
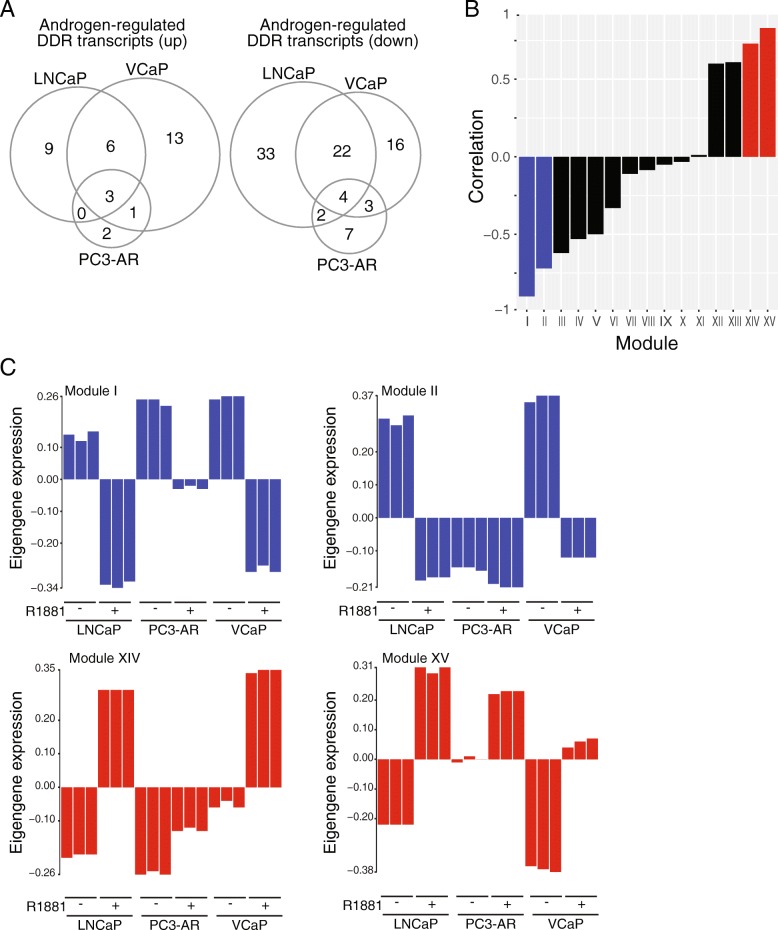
Comparison of DNA damage response gene expression in prostate cancer cell lines. **a** Venn diagram of differentially expressed DNA repair genes in LNCaP, VCaP, and PC3-AR cell line models. A total of 450 expert-curated DNA damage repair genes were assessed for androgen-mediated expression changes (2 nM R1881) using the following cut-offs: > 2-fold or < 0.5-fold; adj. *p* ≤ 0.05. LNCaP and VCaP treatments were conducted for 24 h while PC3-AR data was taken from a 12 h treatment. **b** Gene modules detected from WGCNA of RNA-sequencing. Positive (red) and negative (blue) gene expression correlations were significantly related to androgen treated. **c** Eigengene plots for gene modules I, II, XIV, and XV where gene sets were significantly associated with androgen treatment. Gene lists can be found in Additional file 7: Table S5

Our analysis using the 32-gene set confirms that androgen signaling has a role in regulating DNA repair gene expression, with both positive and negative changes, depending on the gene. This prompted us to expand our analysis, which we did using two approaches. In the first approach, we used the list of differentially expressed genes from all three cell lines and filtered the list to include only those which are part of a set of 450 expert-curated DNA damage response (DDR) [21]. Using this strategy, we found 34 DDR genes that were significantly upregulated (adjusted *p*-value < 0.05) and 87 DDR genes that were significantly downregulated (adjusted *p*-value < 0.05) in response to androgen treatment (Fig. 5a**,** Additional file 5: Table S4). Only seven DDR genes showed androgen-mediated differential expression in all three prostate cancer cell lines. We observed that three DNA repair genes (*CDKN1A*, *NCAPD3*, and *PER1*) were significantly upregulated by androgen treatment in each of the three cell line models, whereas four DDR genes (*EXO1*, *XRCC2*, *PER3*, and *TERT*) were significantly decreased (Fig. 5a). Considering the cell lines as biological replicates and accounting for the differences in the cell lines in the design of the differential expression analysis, we found that 291 of the 450 DDR genes display differential expression in response to androgen (adjusted *p*-value < 0.05), with 192 showing a decrease in expression in response to the treatment (data not shown).

In the second approach, we set out to identify sets of genes (gene modules) that are co-regulated in response to androgen by constructing differential co-expression networks and performing topological analysis. To this end, we employed WGCNA [17, 18] to our RNA-seq dataset requiring a minimum module size of 30 genes and identified 15 modules (Fig. 5b-c**,** Additional file 6: Figure S2). Of note, we found four modules that were significantly related to the androgen treatment status (Point-biserial correlation, adjusted p-value < 0.05); two modules were associated with an increase in expression and two modules were associated with a decrease in expression (Additional file 7: Table S5, Fig. 5b-c, red & blue, respectively).

Using the four modules, we selected the genes with kME value > 0.8 and ranked them based on the direction of the fold-change and p-value. We then used Enrichr [53] to identify enriched annotations for each of the four modules. The modules that showed an increase in expression after androgen treatment were found to be enriched for several important pathways related to prostate cancer (Additional file 8: Table S6). Module XV was found to be enriched for cholesterol biosynthesis, steroid biosynthesis, various transport pathways, with genes found to be targeted by several ETS factors GABP, ERG, and ETS1 (p-adjusted < 0.05). Module XIV was also enriched for similar pathways to Module XV. Moreover, this gene set was significantly enriched for genes reported to be highly expressed in the prostate (BioGPS, p-adjusted 0.002591). Module I and Module II were each associated with a decrease in expression after androgen treatment. Module I was not significantly enriched for a particular pathway, while Module II was significantly enriched for ribosomal genes. But because the enrichment analyses did reveal well-established pathways associated androgen signaling in prostate cells, we believed the approach could be used to determine the presence of DNA repair genes in the modules.

We compared the genes from the modules that were significantly related to androgen treatment (Modules I, II, XIV, XV) to the expert-curated list of 450 human DDR genes [21]. We found 25 genes in modules that were affected by androgen. There were 17 genes in modules that showed an increase in expression after androgen treatment, and eight genes were in modules that showed a decrease after androgen treatment (Additional file 9: Table S7). By examining publicly-available AR ChIP-sequencing data from LNCaP and VCaP cells (GSE28126), we found that 18/25 of these genes have proximal AR binding sites (Additional file 9: Table S7, indicated in red). Interestingly, the only gene shared between the 25-gene set described here and the 32-gene set reported in Polkinghorn et al. (2013) [50] was *HUS1* while five genes in our 25-gene set were identified within a 144-gene set as significantly associated with AR output in human prostate cancer samples. Within our 25 gene set are *EYA3* and *MTOR*, which have been identified as putative AR targets in an integrative study of TCGA-PRAD samples [54]. Our module analysis expands the number of DNA repair genes impacted by androgen signaling, but it also underscores the difficulties of predicting how DNA repair activity is affecting the biological outcome since there are both positive and negative effects on gene expression.

### Advanced prostate cancer exhibits aberrant expression of the DNA repair machinery

Our analyses identified a set of 25 DNA repair genes affected by androgen treatment across three cell lines and other studies have recently identified the importance of this signaling pathway in large cohorts of advanced prostate cancer [55–57]. We examined if our DDR gene set, or any of the 450 expert-curated DDR genes, were associated with prostate cancer disease progression. We analyzed microarray data from cases of primary and metastatic prostate cancer as well xenograft models of castration resistance. We downloaded a Z-score normalized (with respect to the normal prostate samples) expression dataset from the MSKCC study [25] provided by cBioPortal [23, 24]. We restricted the analysis to the 450 DDR genes, and performed a Kruskal-Wallis rank sum test to identify genes that showed significant association (*p*-value <1e-5) with the provided clinical tumor status (primary tumor or metastatic tumor). In total, 42 DDR genes passed the threshold (Additional file 10: Table S8), and the expression levels of these genes help distinguish metastatic versus primary prostate cancer. It has been shown in other studies that DNA repair pathway gene alterations are more frequent in the metastatic samples compared to primary tumors [58], but further analyses are needed to confirm if those alterations lead to expression differences of specific genes and gene sets.

**Fig. 6:**
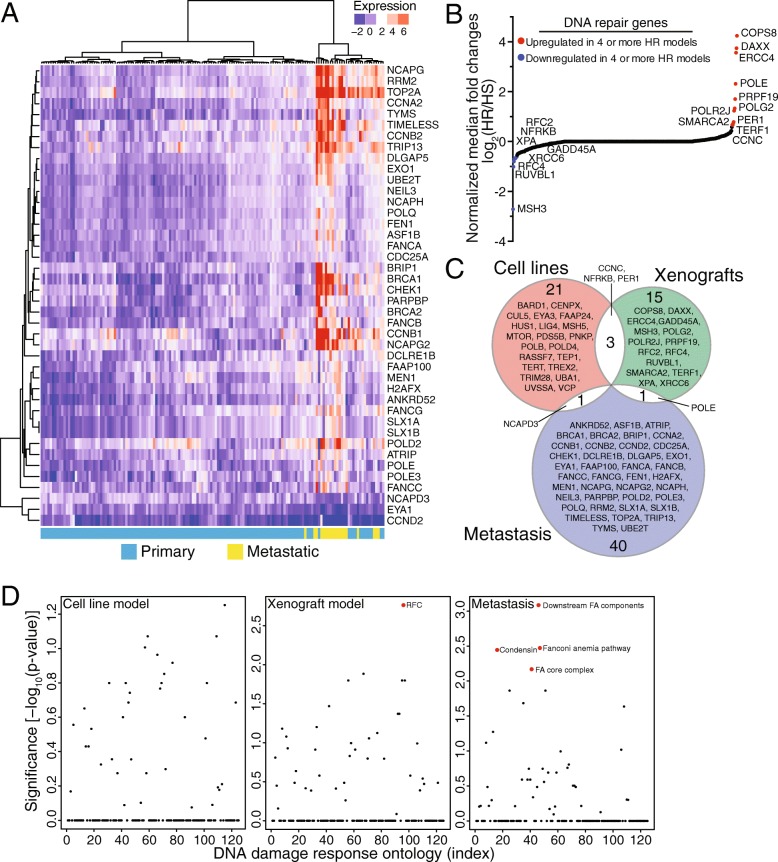
DNA damage response genes are differentially regulated in prostate cancer models and in advanced prostate cancer. **a** Heatmap of Z-score normalized DNA repair genes significantly (Kuskal-Wallis test, *p* ≤ 1E-5) associated with metastatic prostate tumors. Publicly available MSKCC data of 131 primary tumors and 37 metastatic tumors was downloaded from cBioPortal. **b** S-plot of castration-mediated expression changes of 450 DNA damage repair genes. Median fold changes were calculated from 7 hormone-resistant versus hormone-sensitive prostate cancer xenograft models. Data was downloaded and derived from Chen et al. (2004) [20]. Red dots represent genes whereby four or more xenograft models were upregulated while blue dots represent genes whereby four or more xenograft models were downregulated. **c** Venn diagram of DNA repair machinery differentially regulated in the cell line, xenograft, and patient models. **d** Graph of Fisher’s exact test comparing DNA damage response genes from each dataset (cell line, xenograft, and patient metastases) to the 125 ontology groups described by Pearl et al. (2015) [21]. Red dots and descriptions indicate *p* ≤ 0.01

Because we observed that DNA repair gene expression is increased in prostate cancer metastases (Fig. 6a), we tested whether DNA repair genes are altered in models of hormone-resistant (HR) prostate cancer. We used publicly-available gene expression microarray data from seven prostate cancer xenografts (CWR22, LAPC4, LAPC9, LNCaP, LuCaP23, LuCaP35, and LuCaP41) for which there are hormone sensitive (HS) and hormone resistant (HR) isogenic lines [20]. To determine changes in expression levels, we calculated expression fold changes between xenograft tumor samples developed in androgen-deprived conditions and the corresponding control tumor sample. When we considered all 450 DDR genes, we found 19 genes were differentially expressed in at least four of the seven prostate cancer xenograft models (Fig. 6b). More specifically, we found the expression of 11 genes were increased and eight genes were decreased in the HR lines grown in castrated mice (Additional file 10: Table S8).

Integrating the findings from prostate cancer cell lines, patient tumor samples, and xenografts, we found a total of 81 DDR genes display expression changes associated with androgen signaling and disease progression (Fig. 6c). There was very limited overlap between these gene sets. The *PER1*, *NFRKB*, and *CCNC* genes were altered in prostate cell lines and in the HR xenograft models. *POLE* was differentially expressed in the HR xenograft models and in patient metastases. *NCAPD3* was differentially regulated in patient metastases and was regulated by androgen treatment in the cell line models. These 81 genes play important roles in multiple DNA repair pathways through checkpoint signaling, chromatin remodeling, chromatin segregation, and base excision repair.

With the goal of understanding how the 81 DDR genes (Fig. 6c) contribute to specific repair pathways, we used a Fisher’s exact test to compare the 81 DDR gene set to the 125 DNA repair ontologies organized and described by Pearl et al. (2015) [21]. Using a *p*-value cut-off of 0.01, we found there are no ontologies that are common to the cell line models, one ontology that is common to the xenograft models, and four ontologies that are common to the patient model (Fig. 6d). Thus, it appears that the expression alterations to the DNA repair pathway are highly specific to the model and possibly the disease stage. This further underscores the importance of understanding the genomics and epigenomics of the model systems that are frequently used to study prostate cancer.

### Treating prostate cancer cells with the MRE11 inhibitor

Studies from several groups have shown that androgen signaling through AR induces a low level of transcription-associated DNA damage [59, 60]. This might be a characteristic of steroid hormone receptor signaling given that similar observations have been made in cells treated with estrogen and glucocorticoid [61–63]. Thus, it is plausible that androgen-induced expression of DNA repair genes is part of a mechanism to restore the integrity of chromatin that is damaged during transcription. Importantly, the androgen effects on DNA repair also includes androgen and AR-dependent recruitment of DNA repair factors to sites of damage, which have been shown to occur at AR-regulated enhancers and promoters [59, 60, 63]. ATM, DNA-PK, DNA Ligase IV, MRE11, and other repair factors undergo transient recruitment to AR binding sites in an androgen-dependent manner [60, 64, 65]. The application of drugs targeting DNA repair enzymes has helped reveal the importance of DNA factors to AR-dependent transcription [60, 64, 66]. We used a drug approach to test whether the small molecule inhibitor mirin, which targets MRE11 [67], can be used to inhibit AR-dependent transcription. MRE11, an enzyme that has both 3′-5′ exonuclease and endonuclease activity, is a component of the DNA damage sensing MRN complex. Mirin has been characterized extensively in a variety of cellular and biochemical contexts where it inhibits events associated with homologous recombination [67, 68], but to our knowledge, mirin has not been characterized in prostate cancer cells. Thus, we set out to answer two simple questions. (1) Does mirin inhibit AR-dependent transcription in prostate cancer cells? (2) Does mirin inhibit prostate cancer cell growth?

**Fig. 7:**
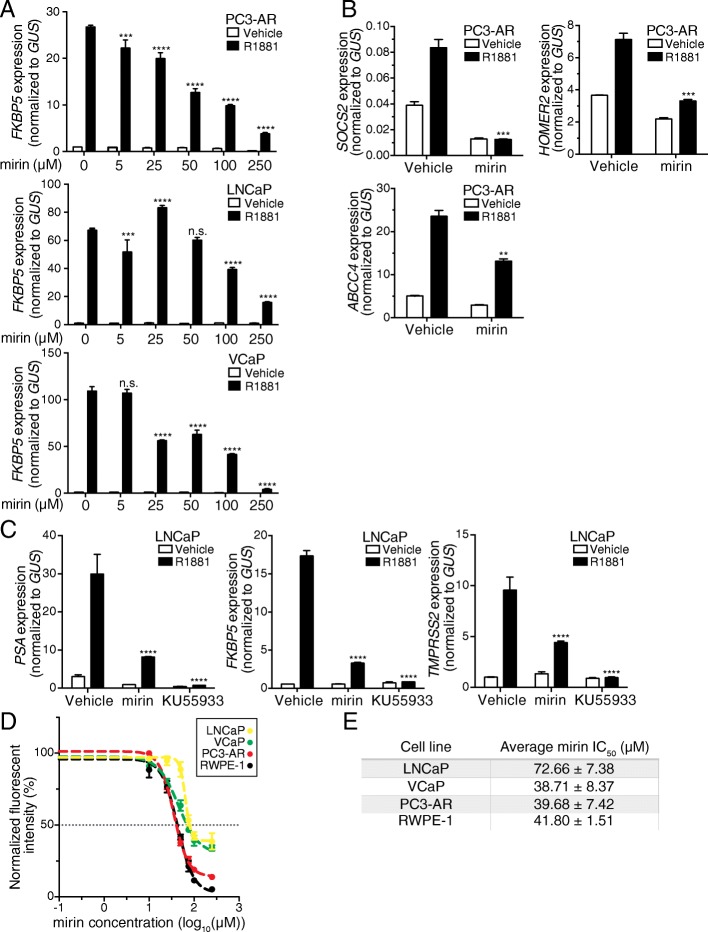
Mirin inhibition of MRE11 reduces androgen-stimulated gene expression. **a** Mirin dose response of androgen-mediated transcription (2 nM R1881, 12 h). Relative expression (RT-qPCR) of *FKBP5* mRNA in LNCaP, VCaP, and PC3-AR cells. **b** Relative expression (RT-qPCR) of *SOCS2*, *HOMER2*, and *ABCC4* in PC3-AR cells. **c** Relative expression (RT-qPCR) of three androgen-mediated genes *PSA*, *FKBP5*, and *TMPRSS2* after inhibiting MRE11 with mirin or ATM with KU55933 in LNCaP cells. **d** Alamar blue cell viability assay measuring mirin effect on cell growth/survival in RWPE-1, LNCaP, VCaP, and PC3-AR after 72 h. **e** Mirin IC_50_ values derived from Alamar blue cell viability assays from three experimental replicates

We tested a broad range of mirin concentrations for effects on androgen-induction of *FKBP5* in PC3-AR, LNCaP, and VCaP cells. We found that 50 μM mirin reduced the androgen-induction of *FKBP5* by 50% (Fig. 7a). LNCaP cells appeared less sensitive to mirin, where 100 μM mirin was required to reduce *FKBP5* by 50% (Fig. 7a). Mirin treatment reduced androgen induction of the *SOCS2*, *HOMER2*, and *ABCC4* genes in PC3-AR cells (Fig. 7b). Partial knockdown of *MRE11* by siRNA generated a modest but statistically significant reduction in the *TMPRSS2* and *PSA* genes in LNCaP cells (Additional file 11: Figure S3A-B). These data, which indicate that mirin can be used to reduce androgen-induced transcription in prostate cancer cells expressing WT and mutant forms of AR, support the model proposed by other groups that DNA repair is important for AR-dependent transcription [64–66]. As we alluded to earlier, ATM is also recruited to sites of androgen-induced damage [60, 65], and is, in part, regulated in part by MRE11 [69]. Application of the ATM inhibitor KU55933 [70] caused a striking reduction in androgen induction of the *PSA*, *FKBP5*, and *TMPRSS2* genes in LNCaP cells (Fig. 7c). These show that inhibition of the DNA repair machinery can be used as a strategy to reduce androgen signaling in prostate cancer cells, and support the view that DNA repair makes an important contribution to AR-dependent transcription.

To examine the effect of mirin on prostate cancer cell growth and survival, we treated cells with drug for 72 h and performed an Alamar blue assay. We determined that PC3-AR, VCaP, RWPE-1, and LNCaP cells all display IC_50_ values in the range of ~ 40–70 μM (Fig. 7d-e). LNCaP cells appeared to be somewhat less sensitive to mirin than the other cell types, a result noted in the transcription assays (Fig. 7a). Importantly, mirin antagonism of androgen signaling does not result in the accumulation of damaged DNA, at least assessed by immunoblotting for global changes in activated ATM and γH2AX (Additional file 11: Figure S3C).

## Discussion

The discovery that PARP inhibitors have efficacy in prostate cancer patients that harbor mutations in DNA repair genes provides proof-of-concept that targeting the DNA repair machinery can be beneficial. Defining the genomic status and RNA expression of the DNA repair machinery in prostate cancer cell lines provides a knowledge base that is critical for the selection of an appropriate model, and interpretation of data generated from the model. This could include evaluating the effects of clinically-used drugs, screening for new compounds, and searching for potential synthetic interactions. In this study, we set out to understand the genetic changes in the DNA repair machinery in LNCaP, VCaP, PC3-AR, and RWPE-1 cell lines. We found a total of 24 small nucleotide variants with low allelic frequency across these prostate cancer cell lines. Mutations were detected in the following genes: *ATM*, *ATR*, *BRCA1*, *BRCA2*, *CHEK2*, *PRKDC*, *ERCC3*, *FANCA*, *HDAC2*, *MLH3*, *NBN*, *PARP1*, and *RAD50*. Of these 24 mutations, two mutations in LNCaP cells are predicted to be deleterious CHEK2(E239*) and RAD50(L719 fs*15). The *RAD50* mutation (chr5:131931452; L719 fs*15) causes a frameshift and a large C-terminal truncation that results in loss of > 500 amino acids. The *CHEK2* mutation (chr22:29107974; E239*) introduces a stop codon that deletes the kinase domain. CHEK2 activation in response to DNA damage induces a cell cycle checkpoint [71]. *CHEK2* variants predispose individuals to breast and colon cancer [72] and it has been shown to be a negative regulator of prostate cancer growth [73]. RAD50 is a member of the MRN (MRE11-RAD50-NBS1) complex which functions as a scaffold for sensing DNA damage [74]. Mouse knockouts of *RAD50* are embryonic lethal [75], and, like *CHEK2*, *RAD50* mutations are associated with cancer risk [76, 77]. Mutations in *CHEK2* or *RAD50* could help sensitize LNCaP cells to DNA damage induced by chemotherapy drugs and IR. From sequence data, the genomic alterations in *CHEK2* and *RAD50* appear to affect single alleles in LNCaP cells, but *CHEK2* and *RAD50* are both haploinsufficient genes, the level of activity provided from a single WT allele may not provide enough activity for the cell.

Additionally, we detected missense mutations in two additional DNA repair genes that are predicted to impact protein function. The *ERCC3* mutation (chr2:128044450; R391W) introduces a tryptophan into the Helicase C-terminal domain, and the *PARP1* mutations (chr1:226566948; E547G and chr1:226555302; V762A) introduce amino acid changes into functional domains of the protein. One of the amino acid changes, V762A, has been studied biochemically and shown to reduce PARP-1 enzyme activity [78, 79].

Our characterization of DNA repair genes in prostate cancer cells included generating RNA-seq data, determining the extent to which DNA repair genes are regulated by androgen in different cell lines, and using existing data sets to make comparisons with xenograft models and human prostate cancer. Androgen signaling through AR has been shown to induce a DNA repair signature (32 genes) that could explain the radioresistance of some prostate cancers [50].

Using RNA-seq data and WGCNA, we found that 17 DNA repair genes were positively correlated with androgen treatment across three cell lines, and eight DNA repair genes were negatively correlated with androgen treatment. Surprisingly, only one member of the 32 DNA repair gene set reported by Polkinghorn et al. (2013) [50], *HUS1*, was detected in our 25 DNA repair gene set. The differences could be due to any number of biological variables associated with the cell lines or growth conditions in laboratories as our experiments were conducted using shorter R1881 treatment times.

It is possible that cell cycle changes pertaining to the observed androgen-induced G1 cell cycle arrest could contribute to the gene expression profiles detected in response to androgen - particularly because our analysis shows that all three cell lines displayed a strong androgen-regulated decrease in the E2F gene target hallmark (Fig. 3d). The E2F transcription factor family plays important roles as both activators and repressors of the cell cycle [80]. Mechanistically, both transcription factors – AR and E2F1 – could contribute either cooperatively or independently to the changes in DNA damage response genes as has been shown previously for other androgen-regulated genes [81].

Ultimately, our findings clearly support a role for androgen signaling in DNA repair gene expression in multiple cell lines, xenograft models, and in human tumor samples. As part of the analysis, we found that 60% of the DNA repair genes affected by androgen treatment in cell lines have AR binding sites based on AR ChIP-seq data from LNCaP and VCaP cells [44]. The implication from our data is that androgen regulation of DNA repair gene expression could influence the response of prostate cancers to radiotherapy. But because there are both positive and negative effects on DNA repair gene expression, and there exists a complex interplay between DNA repair pathways, it is difficult to make simple predictions as to the biological outcome.

We also observed that DNA repair gene expression is changed during the transition from androgen-dependent to castrate-resistant cell growth. We examined publicly available microarray data from “HS-HR” xenograft pairs [20] and found a total of 19 DNA repair genes were up- or down-regulated in at least four (of seven) models for CRPC. Using patient data, we found a set of 42 DNA repair genes were associated with metastasis. Some of these DNA repair genes have AR binding sites within 25 kb of the transcriptional start site or within the gene body itself, suggesting the regulation of a subset of these gene could be directly regulated through androgen signaling. The very small overlap between these sets of DNA repair genes might be explained by the fact each was generated from cells grown in vastly different milieu and selection pressures (cell culture, xenograft, human tumors). Genes enriched for specific pathways, namely cell growth and cell cycle, have been shown to be uniquely regulated in cell lines versus patient tumor samples [82].

While the DNA repair machinery is widely appreciated for its role in correcting mutations generated during replication, and in response to various environmental insults, there is growing acceptance of its importance in transcription. The introduction of DNA breaks, for the purpose of resolving topological constraints [59, 60, 63] and for expression of enhancer RNAs [65], has been shown to be important for AR-dependent gene expression. Not surprisingly, androgen-dependent induction of these DNA breaks is accompanied by recruitment of DNA repair components, which occurs on a time scale of minutes. This mechanism might explain why certain DNA repair inhibitors reduce androgen-stimulated gene expression. Topoisomerase-mediated DNA breaks have been shown to be necessary and sufficient for transcription of genes that direct early events in neuronal differentiation [83].

One of the DNA repair enzymes that was shown by the Rosenfeld group to be important for androgen-induced gene expression is MRE11, which is a component of the MRN complex [65]. Mutation of *MRE11* was reported within the cohort of prostate cancer patients that showed a positive response to Olaparib [6]. These findings led us to test whether the MRE11 inhibitor, mirin, can be used to inhibit AR-dependent transcription and prostate cancer growth. Mirin inhibition of growth in non-prostate cell types also required relatively high concentration of drug, with IC_50_ values ranging from 12.5–100 μM [67, 84–87]. This is comparable to the values we obtained in prostate cancer cells (Fig. 7e-f). We found that mirin inhibits transcription of multiple AR target genes in PC3-AR, LNCaP, and VCAP cells. The finding that androgen-induced expression of *FKBP5* was inhibited by mirin in all three cell lines argues that MRE11 complex function is critical for transcription in prostate cancer cells, which is consistent with the siRNA results published by another group [65]. Androgen and mirin, used alone and in combination, did not have a noticeable effect on ATM levels, ATM activation, or H2AX phosphorylation. Thus, the mirin inhibition of AR-dependent transcription and cell growth is not accompanied by a global change in DNA damage signaling.

## Conclusions

In summary, we have identified several deleterious mutations in the DNA repair machinery, presented evidence that expression of DNA repair enzymes is impacted by androgen signaling, and shown that small molecule inhibition of a DNA repair enzyme is useful for inhibiting AR-dependent gene expression. Our analysis suggests there might not be a simple DNA repair enzyme signature associated with androgen signaling and prostate cancer progression that is shared between models and patients. The data does, however, underscore the important interplay between androgen signaling and the DNA damage response and reinforces the notion that targeting DNA repair enzymes can be a useful approach to inhibit prostate cancer cells.

## Additional files

Additional file 1: **Table S1.** DNA damage response gene variants in prostate cancer cell lines. (XLSX 16 kb)

Additional file 2: **Table S2.** Potential deleterious alterations of DNA damage response genes in prostate cancer cell lines. (XLSX 15 kb)

Additional file 3: **Table S3.** GSEA molecular hallmarks of the gene expression modules from WGCNA of PC3-AR data. (XLSX 19 kb)

Additional file 4: **Figure S1.** Standard GSEA plots of the HALLMARK_ANDROGEN_RESPONSE gene set. Analysis included pre-ranked androgen-mediated expression changes for LNCaP, VCaP, and PC3-AR cell lines. (PDF 933 kb)

Additional file 5: **Table S4.** DNA damage response genes differentially regulated by androgen associated with Fig. 5a. (XLSX 10 kb)

Additional file 6: **Figure S2.** Gene modules detected from WGCNA of RNA-sequencing. Modules III through XIII were not significantly related to androgen treated. (PDF 485 kb)

Additional file 7: **Table S5.** All genes from WGCNA associated with androgen treatment (Modules I, II, XIV, and XV). (XLSX 45 kb)

Additional file 8: **Table S6.** Top 10 WikiPathways for the gene sets from Modules I, II, XIV, and XV determined by Enrichr. (XLSX 11 kb)

Additional file 9: **Table S7.** DNA damage response genes associated with androgen treatment in prostate cancer cell lines determined by WGCNA. (XLSX 9 kb)

Additional file 10: **Table S8.** DNA damage response genes in prostate cancer xenografts and patient metastases. (XLSX 10 kb)

Additional file 11: **Figure S3.** Androgen-stimulated gene expression is inhibited with MRE11 knockdown and mirin treatment does not induce widespread DNA damage. (**A**) Immunoblot showing MRE11 knockdown in LNCaP cells. (**B**) Androgen-mediated transcription is inhibited with *MRE11* knockdown. Relative expression (RT-qPCR) measuring transcription of *PSA* and *TMPRSS2*. (**C**) Immunoblot of phospho-ATM (S1981), ATM, γH2AX, and H2AX levels in response to mirin treatment. Androgen-treated LNCaP cells were incubated for 6 h with 10 nM R1881. (PDF 2812 kb)

## Abbreviations

ADT: Androgen deprivation therapy
AR: Androgen receptor
CRPC: Castration resistant prostate cancer
DDR: DNA damage response
SNV: Single nucleotide variant
