# Supplementary figures and images for: Genomic analysis of DNA repair genes and androgen signaling in prostate cancer
Source: BMC Cancer. 2018 Oct 10;18:960. doi: 10.1186/s12885-018-4848-x (PMC6180441; doi:10.1186/s12885-018-4848-x)

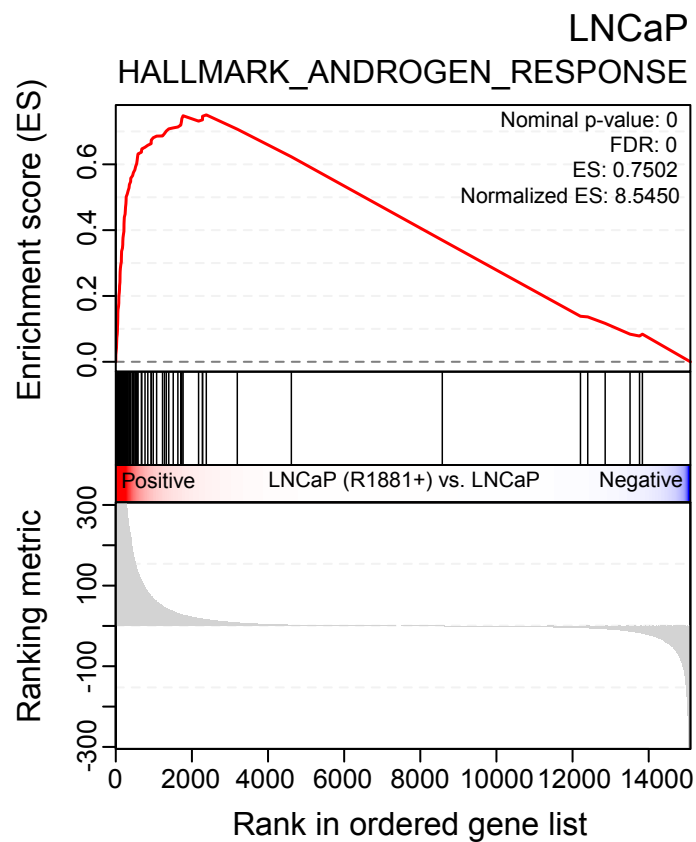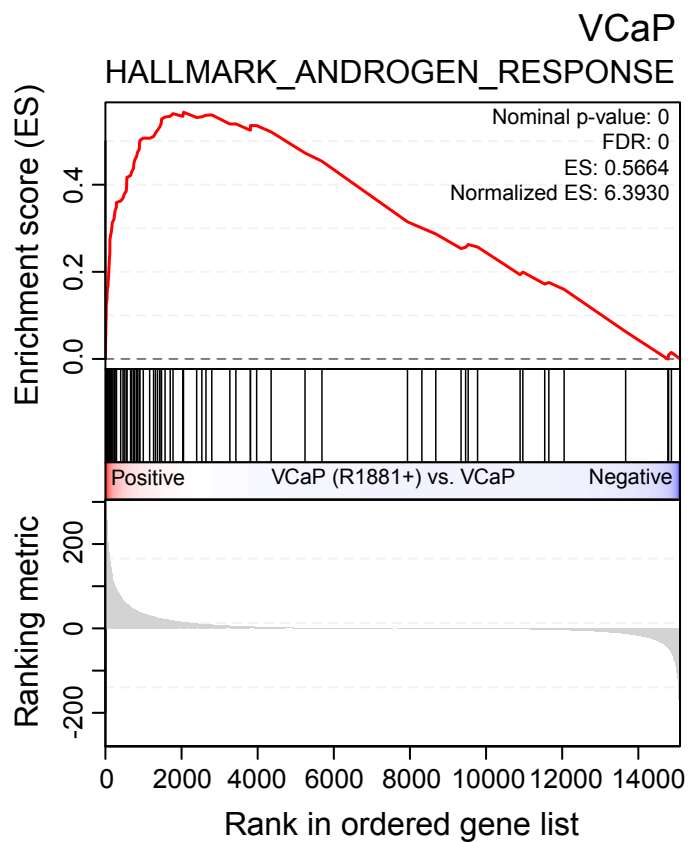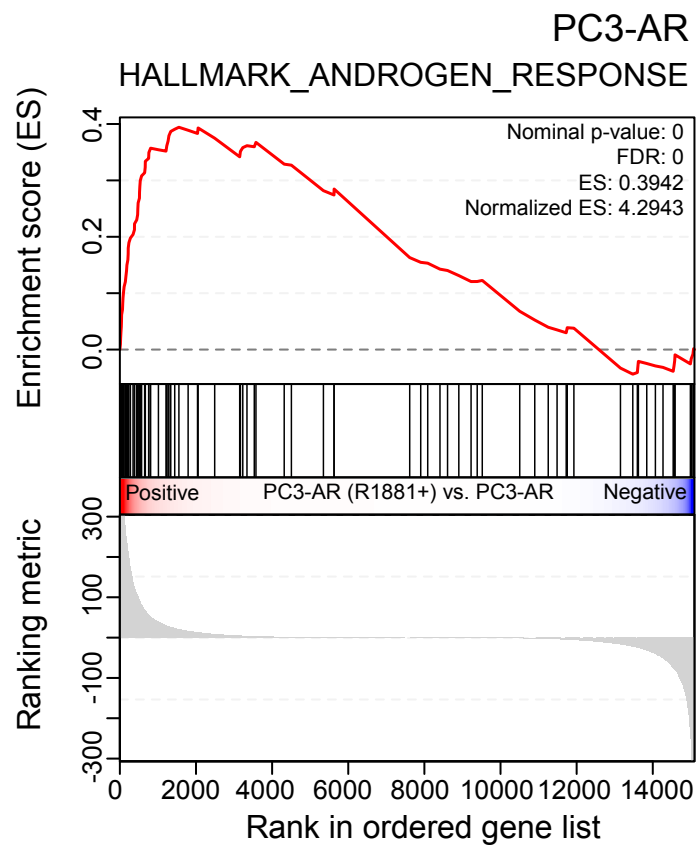

Supplement: Supplementary file 4 — Figure S1. Standard GSEA plots of the HALLMARK_ANDROGEN_RESPONSE gene set. Analysis included pre-ranked androgen-mediated expression changes for LNCaP, VCaP, and PC3-AR cell lines. (PDF 933 kb) [file 12885_2018_4848_MOESM4_ESM.pdf]

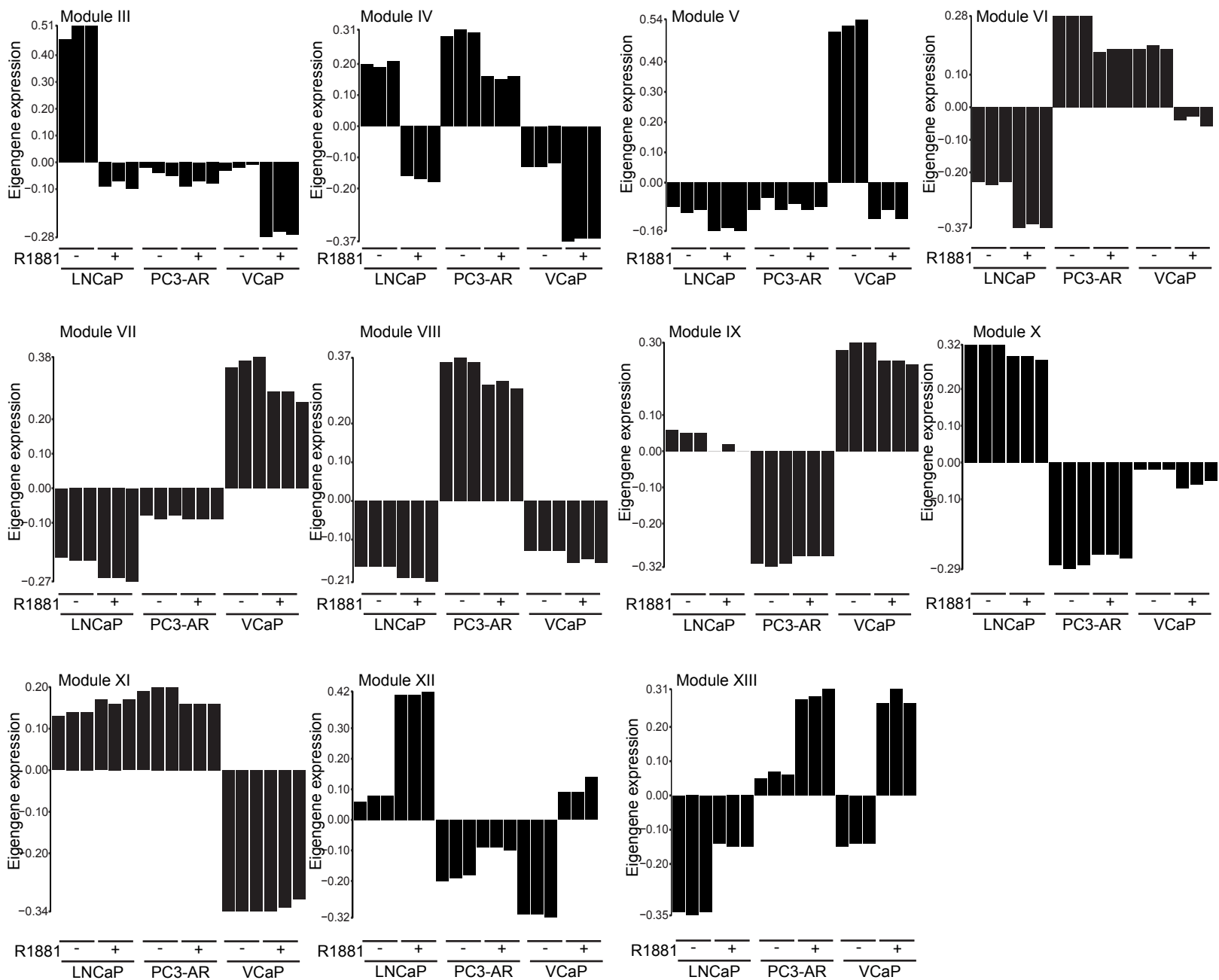

Supplement: Supplementary file 6 — Figure S2. Gene modules detected from WGCNA of RNA-sequencing. Modules III through XIII were not significantly related to androgen treated. (PDF 485 kb) [file 12885_2018_4848_MOESM6_ESM.pdf]

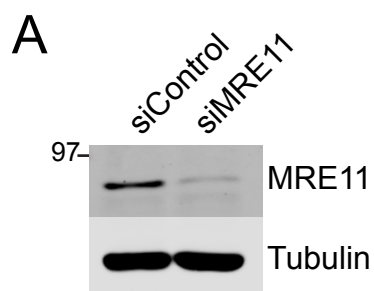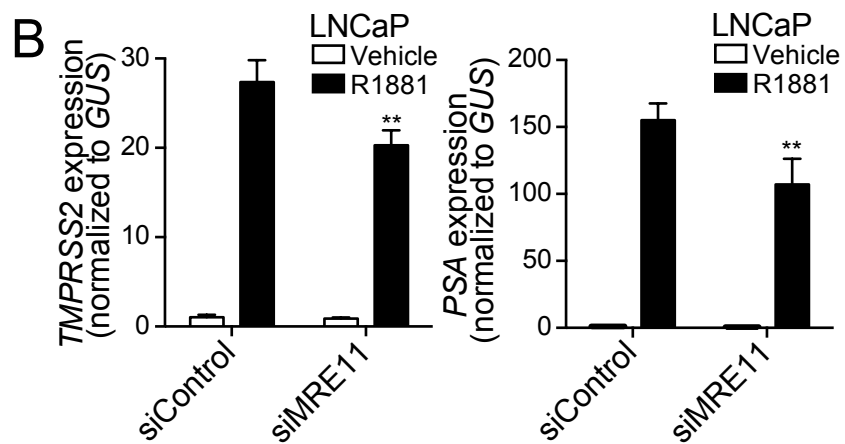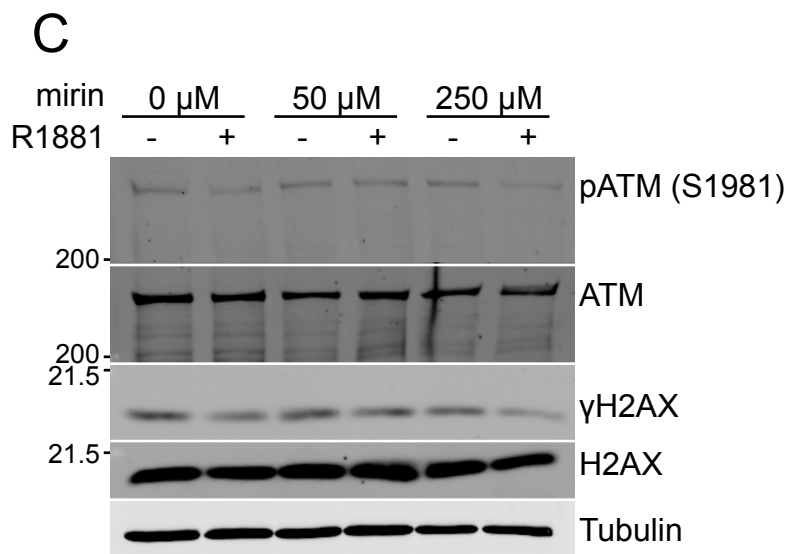

Supplement: Supplementary file 11 — Figure S3. Androgen-stimulated gene expression is inhibited with MRE11 knockdown and mirin treatment does not induce widespread DNA damage. (A) Immunoblot showing MRE11 knockdown in LNCaP cells. (B) Androgen-mediated transcription is inhibited with MRE11 knockdown. Relative expression (RT-qPCR) measuring transcription of PSA and TMPRSS2. (C) Immunoblot of phospho-ATM (S1981), ATM, γH2AX, and H2AX levels in response to mirin treatment. Androgen-treated LNCaP cells were incubated for 6 h with 10 nM R1881. (PDF 2812 kb) [file 12885_2018_4848_MOESM11_ESM.pdf]
